# Multi-plasticity synergy with adaptive mechanism assignment for training spiking neural networks

**DOI:** 10.3389/fnins.2026.1939650

**Published:** 2026-09-09

**Authors:** Zhibin Li, Haiteng Wang, Yuzhe Liu, Chenxiang Ma, Susanna Gordleeva, Qiang Yu

**Affiliations:** 1 School of Artificial Intelligence, College of Intelligence and Computing, Tianjin University, Tianjin, China; 2 Institute of Neurosciences, National Research Lobachevsky State University of Nizhny Novgorod, Nizhny Novgorod, Russia; 3 Institute for Embodied Intelligence, Tianjin University, Tianjin, China

**Keywords:** hybrid learning, multi-plasticity synergy learning, spiking neural networks, supervised learning, synaptic plasticity, unsupervised learning

## Abstract

Spiking Neural Networks (SNNs) are promising brain-inspired models known for low power consumption and superior potential for temporal processing, but identifying suitable learning mechanisms remains a challenge. Despite the presence of multiple coexisting learning strategies in the brain, current SNN training methods typically rely on a single form of synaptic plasticity, which limits their adaptability and representational capability. In this paper, we propose a biology-motivated computational framework that incorporates multiple synergistic plasticity mechanisms for more effective SNN training. The framework is inspired by the coexistence of heterogeneous regulatory and plasticity-related processes in biological neural systems, rather than directly corresponding to specific biological learning rules. Our method enables diverse learning algorithms to cooperatively modulate the accumulation of information, while allowing each mechanism to preserve its own relatively independent update dynamics. We evaluated our approach on diverse datasets to demonstrate that our framework significantly improves performance and robustness compared to conventional learning mechanism models. This work provides a general and extensible foundation for developing more powerful SNNs guided by multi-strategy brain-inspired learning.

## 1 Introduction

Spiking neural networks (SNNs) have attracted increasing attention because of their event-driven computation, strong temporal processing capability, and high energy efficiency (Roy et al., 2017; Kasabov et al., 2013; Kim et al., 2020; Yu et al., 2020; Yao et al., 2023), making SNNs a promising alternative to traditional artificial neural networks (ANNs). By incorporating bio-inspired neuronal dynamics (Maass, 1997; Ros et al., 2006; Deco et al., 2008), SNNs offer a solid foundation for brain-inspired computation and low-power neuromorphic systems. However, effective SNN training remains a fundamental challenge in practice. The discrete nature of spikes, the non-linear evolution of membrane potentials, and the long-range temporal dependencies in neural responses together make optimization substantially more difficult than in conventional ANNs.

To address these challenges, a variety of learning methods have been proposed for SNNs, including gradient-based methods such as Spatio-temporal Backpropagation (STBP) (Wu et al., 2018) and Backpropagation Through Time (BPTT) (Lee J. H. et al., 2016), biologically inspired local plasticity rules such as Spike-Timing-Dependent Plasticity (STDP) (Kheradpisheh et al., 2018), and more recent alternatives such as self-backpropagation (SBP) (Zhang et al., 2021) and Hybrid Global-Local Learning (HGLL) (Wu et al., 2022). These approaches have shown distinct strengths across different tasks and learning settings. For example, gradient-based plasticity mechanisms are effective for global error-driven optimization, whereas local plasticity mechanisms are better suited for capturing correlation structures and supporting more biologically plausible adaptation. However, most existing approaches consider single plasticity mechanisms or limited combinations of plasticity mechanisms, largely overlooking the potential for synergistic interaction among multiple plasticity mechanisms. This limitation makes it difficult to simultaneously capture complex neuronal dynamics, extract rich and comprehensive features, and maintain strong generalization and robustness across diverse tasks.

In contrast, biological learning is not governed by a single mechanism. Instead, neural adaptation often involves multiple regulatory processes acting together across different temporal and functional scales, with interactions between plasticity mechanisms at different timescales shaping neural representations (Kandel, 1991; Edelmann et al., 2017; Gonzalez et al., 2025; El-Sayes et al., 2019; Yu et al., 2025). At the system level, the coexistence and coordinated interaction of multiple plasticity mechanisms–such as Hebbian STDP, homeostatic plasticity, and neuromodulatory regulation–have been widely observed in biological neural circuits. Separate processes contribute distinct roles to adaptation, and coordinated interaction among such processes is common in biological systems. This observation suggests that effective SNN training can benefit from the coordinated use of multiple learning mechanisms with complementary roles.

**Figure 1:**
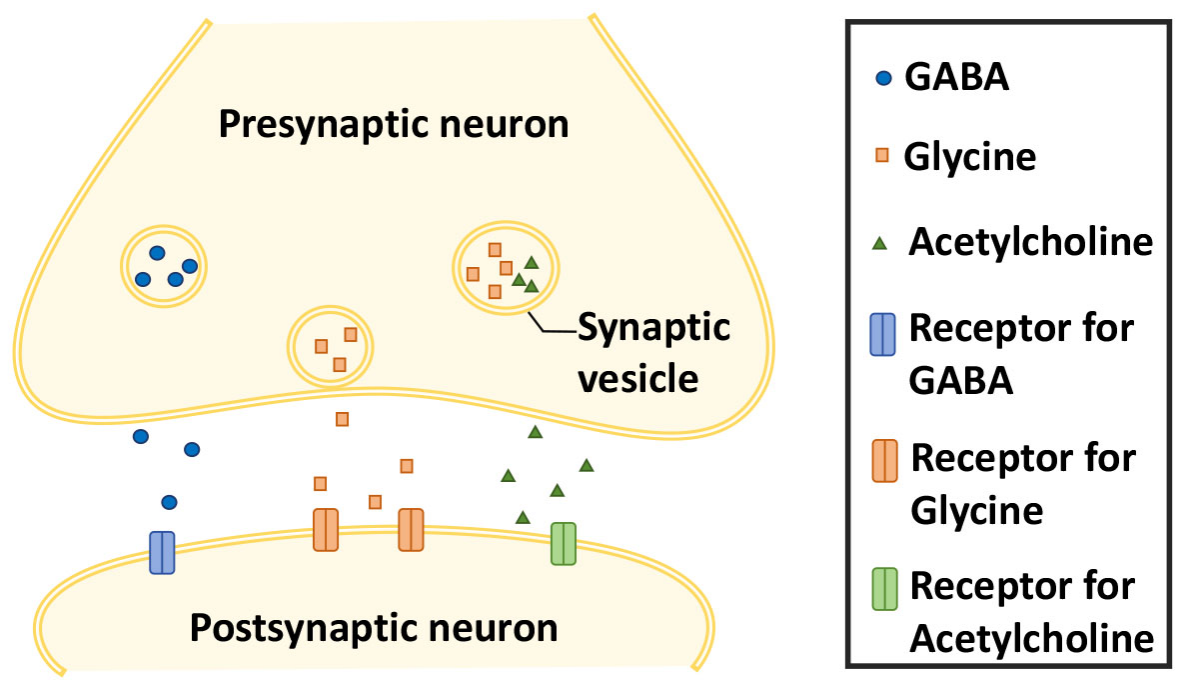
Organizational analogy of functionally differentiated parallel signals jointly shaping a common neural response. A pre-synaptic neuron releases different neurotransmitters into the synaptic cleft, and each neurotransmitter acts on corresponding receptors of the post-synaptic neuron through a distinct pathway. Such parallel pathways remain functionally differentiated while jointly influencing post-synaptic responses. This schematic illustrates an organizational principle of multi-pathway coordination and does not imply that the depicted neurotransmitter pathways correspond to specific learning rules. The neurotransmitter types shown here are schematic and vary across cell types.

A related organizational principle is illustrated by neurotransmitter co-release, where several regulatory influences act on the same neural activity through different pathways (Jaim-Etcheverry, 1994; Gutierrez and Arias-Montano, 2008; Hnasko and Edwards, 2012; Vaaga et al., 2014). As conceptually illustrated in Figure 1, multiple pathways operate concurrently and jointly regulate neural responses under diverse stimuli (Lee S. et al., 2016; Liang et al., 2025). This phenomenon reveals an organizational pattern: parallel influences can remain functionally distinct while jointly shaping a unified adaptive outcome (Bloomfield and Dacheux, 2001; Tritsch et al., 2016; Rozycka and Liguz-Lecznar, 2017). We use neurotransmitter co-release here as an organizational analogy for functionally differentiated signals jointly influencing a common neural response, rather than as biological evidence that multiple learning rules operate independently at the same synapse.

Motivated by this system-level principle, we propose a Multi-Plasticity Synergistic Learning (MPSL) method for SNNs. Instead of restricting synaptic adaptation to a single learning paradigm, MPSL provides a unified learning formulation in which diverse learning mechanisms with complementary strengths collaboratively guide membrane potential accumulation while preserving their distinct update behaviors. To flexibly coordinate their contributions, we introduce adaptive learnable modulation coefficients during the fusion process. In this way, the proposed method enables heterogeneous plasticity signals to interact synergistically within a single computational model, with the goal of improving performance, generalization, and robustness. Importantly, MPSL is not tied to any specific set of learning rules and maintains general applicability to a broad class of plasticity mechanisms. For empirical validation, we instantiate the proposed method using three representative learning mechanisms, namely STBP, Hebbian learning, and SBP.

Overall, our main contributions are threefold:

- We propose MPSL, a biology-motivated computational framework for spiking neural networks, inspired by the system-level coexistence and coordinated interaction of heterogeneous plasticity-related processes in biological learning. The proposed method enables multiple learning mechanisms to collaboratively guide membrane potential accumulation while preserving their distinct update behaviors.
- We introduce adaptive learnable modulation coefficients to dynamically balance the contributions of different learning mechanisms during the fusion process, enabling effective coordination among heterogeneous plasticity signals within a unified learning scheme.
- We conduct extensive experiments on EEG, image, and speech datasets, together with additional robustness evaluations under noise and cropping perturbations. The results show that the proposed method generalizes effectively across multiple data modalities and consistently enhances both the accuracy and robustness of spiking neural networks.

## 2 Related works

### 2.1 Supervised learning in SNNs

Supervised learning has been a critical driving force in the advancement of SNNs, enabling them to achieve competitive performance across a range of tasks. Unlike traditional ANNs which use continuous activation functions, SNNs rely on discrete spike events, introducing non-differentiable activation dynamics that challenge gradient-based optimization. To address this, early works such as SpikeProp (Bohte et al., 2002) and Tempotron Gütig and Sompolinsky (2006) proposed heuristic gradient-like update rules based on spike timing, providing foundational methods for supervised learning in the temporal domain. More recent methods adopt surrogate gradients, which approximate the non-differentiable spike function with smooth alternatives, thereby enabling BPTT. For example, STBP (Wu et al., 2018) applies BPTT to SNNs by propagating gradients through both spatial layers and temporal membrane states, forming the backbone of modern supervised SNN training. Neftci et al. (2019) further generalized this approach into a unified framework, establishing surrogate gradient learning as a scalable computational solution for optimizing SNNs with standard backpropagation techniques. However, the dependence on explicit labels and global feedback limits their alignment with biological learning and applicability in unlabeled environments. These limitations highlight the need for complementary mechanisms.

### 2.2 Unsupervised learning in SNNs

Unsupervised learning plays a fundamental role in SNNs, often implemented through local synaptic plasticity based on the Hebbian principle of temporal correlation (neurons that fire together wire together) (Hebb, 1949). Building on Hebb's seminal correlation principle, Oja (1982) introduced weight normalization to stabilize synaptic growth, albeit with reduced temporal sensitivity. Subsequent work explored temporal causality through STDP (Markram et al., 1997; Bi and Poo, 1998), which leveraged precise pre-post spike intervals to enhance learning dynamics. Bienenstock et al. (1982) further improved robustness by incorporating dynamic thresholds, though requiring manual parameter calibration. Gütig (2016) extended these principles by integrating subthreshold membrane potentials, bridging discrete spiking events with continuous neuronal dynamics at the cost of implementation complexity. Mozafari et al. (2018) advanced task-driven adaptation through neuromodulatory signals, enabling more flexible plasticity rules. Despite their biological plausibility, these unsupervised methods often suffer from limited task adaptability. In this work, we incorporate Hebbian plasticity into a multi-mechanism learning framework to enhance temporal representation while preserving local learning characteristics.

### 2.3 Hybrid learning in SNNs

Grounded in the three-factor learning theory (Frémaux and Gerstner, 2016; Gerstner et al., 2018)—which integrates pre-synaptic activity, post-synaptic activity, and neuromodulatory signals—these approaches reinterpret global supervision (e.g., task-specific errors or rewards) as biologically plausible third factors modulating synaptic plasticity. Early theoretical foundations for plasticity optimization (Bengio et al., 1992) laid the groundwork for gradient-based supervision of local learning rules. Subsequent work extended these principles to non-spiking architectures, demonstrating broad applicability in meta-learning and dynamic adaptation (Munkhdalai and Trischler, 2018; Miconi et al., 2018). Further innovations automated unsupervised rule through meta-optimization (Metz et al., 2018), collectively advancing hybrid plasticity frameworks across learning paradigms. Wu et al. (2022) integrated a brain-inspired Global-Local learning paradigm with a parametrized spiking differential dynamics model, facilitating efficient hybrid learning on neuromorphic hardware. In parallel, Zhang et al. (2021) established local feedback loops that approximate gradient descent without explicit error propagation, enabling coordinated synaptic modifications across neuron layers and significantly reducing computational costs. Their framework effectively integrates both STDP and SBP mechanisms, demonstrating applicability to both artificial and spiking neural networks. While prior hybrid approaches have explored integrating global and local learning rules, they are often limited to specific combinations or two-way interactions. In contrast, our work introduces a general framework that enables the coordinated use of multiple learning mechanisms, facilitating more flexible multi-mechanism training in SNNs.

## 3 Proposed method

### 3.1 Spiking neuron

We first introduce the Leaky Integrate-and-Fire (LIF) neuron model and its iterative form implemented with the Euler method (Qiu et al., 2024). In a standard LIF neuron, spike generation is determined by the membrane potential and a firing threshold:


$$\begin{array}{l} S^{t,l}=\Theta \left(U^{t,l}-V_{\text{th}}\right)=\{\begin{array}{cc} 1, & U^{t,l}\geq V_{\text{th}}\\ 0, & U^{t,l}<V_{\text{th}} \end{array}, \end{array}$$ (1)

where *S*^*t,l*^ denotes the spike output at time step *t* in the *l*-th layer, *U*^*t,l*^ denotes the membrane potential, and *V*_th_ is the firing threshold.

The synaptic input of a standard spiking neuron is given in Equation 2 as


$$\begin{array}{l} I^{t,l}=f\left(W^{l},S^{t,l-1}\right), \end{array}$$ (2)

where *I*^*t,l*^ is the input current at time step *t* in the *l*-th layer, *W*^*l*^ is the synaptic weight matrix between two adjacent layers, and *f*(·) denotes the synaptic operation. Based on the input current, the membrane potential evolves as


$$\begin{array}{l} U^{t,l}=\rho_{\text{m}}\left(U^{t-1,l}-S^{t-1,l}V_{\text{th}}\right)+I^{t,l}, \end{array}$$ (3)

where ρ_m_ is the membrane decay factor. A soft-reset strategy is adopted in this work. Once a spike is emitted, the threshold voltage is subtracted from the membrane potential.

The above formulation defines the basic spiking dynamics used in this work. Based on this neuron model, different learning mechanisms can be introduced through the synaptic weights, and the coordinated integration of such mechanisms will be described in the following subsections.

### 3.2 Synaptic plasticity

The proposed method is not restricted to a specific set of learning mechanisms. In principle, different plasticity rules can be incorporated as separate update channels. In this work, we instantiate the proposed method with three representative mechanisms, namely STBP, Hebbian plasticity, and SBP, whose complementary learning properties make them suitable for this study. STBP provides global error-driven temporal credit assignment, Hebbian plasticity captures local correlation-based adaptation, and SBP introduces a local-feedback learning signal. Together, the three mechanisms cover distinct yet complementary forms of synaptic adaptation, which makes them suitable for evaluating the effectiveness of the proposed multi-plasticity synergy method. The three mechanisms are introduced separately in this subsection, and their coordinated integration will be described in the next subsection.

#### 3.2.1 STBP-based gradient plasticity

STBP, originally proposed in Wu et al. (2018), provides the error-driven learning channel in the proposed method. Since the spike function in Equation 1 is non-differentiable, direct backpropagation cannot be applied to SNNs. To address this issue, surrogate gradients are used to approximate the derivative of spike generation during backpropagation:


$$\begin{array}{l} \frac{\partial S^{t,l}}{\partial U^{t,l}}\approx u^{\prime }\left(U^{t,l},V_{\text{th}}\right), \end{array}$$ (4)

where *u*′(·) denotes the surrogate gradient function. Following Ding et al. (2024), we use a rectangular surrogate gradient:


$$\begin{array}{l} u^{\prime }\left(U^{t,l},V_{\text{th}}\right)=\frac{1}{a}\mathrm{sign}\left(|U^{t,l}-V_{\text{th}}|<\frac{a}{2}\right), \end{array}$$ (5)

where *a* controls the width of the rectangular window and is set to 1 in all experiments.

With this approximation, the global weight $W_{1}^{l}$ is optimized by STBP over the unfolded spiking dynamics, as given in Equation 6:


$$\begin{array}{l} \frac{\partial L}{\partial W_{1}^{l}}=\underset{t}{\sum }\frac{\partial L}{\partial S^{t,l}}\frac{\partial S^{t,l}}{\partial U^{t,l}}\frac{\partial U^{t,l}}{\partial W_{1}^{l}}\approx \underset{t}{\sum }\frac{\partial L}{\partial S^{t,l}}u^{\prime }\left(U^{t,l},V_{\text{th}}\right)\frac{\partial U^{t,l}}{\partial W_{1}^{l}}, \end{array}$$ (6)

where L denotes the training loss. In this way, $W_{1}^{l}$ receives global error information and performs temporal credit assignment through gradient-based learning.

#### 3.2.2 Hebbian plasticity

Hebbian plasticity provides a local correlation-driven learning channel. Unlike STBP, Hebbian learning does not depend on global error backpropagation. Instead, synaptic change is determined by the interaction between pre-synaptic and post-synaptic activity.

Following the hybrid plastic formulation in Wu et al. (2022), local plasticity is modeled as a continuous synaptic dynamics that consists of two parts: a recovery term that stabilizes the synaptic state, and an activity-driven term that describes local plastic change. In this view, the synaptic update can be written as a diffusion-like process,


$$\begin{array}{l} \tau_{\text{w}}\frac{\text{d}W^{l}}{\text{d}t}=-W^{l}+\eta^{l}S^{t,l-1}\left(\rho \left(U^{t,l}\right)+\beta^{l}\right), \end{array}$$ (7)

where the first term describes the recovery of the synaptic state, and the second term represents Hebbian local plasticity driven by pre-synaptic spikes and post-synaptic activity. Here, *S*^*t,l*−1^ denotes the pre-synaptic spike output, ρ(*U*^*t,l*^) is a bounded non-linear function of the post-synaptic membrane potential, η^*l*^ is the local learning rate, and β^*l*^ is a sliding threshold used to control the direction and magnitude of synaptic change.

By discretizing Equation 7, the Hebbian synaptic weight $W_{2}^{l}$ is updated as


$$\begin{array}{l} W_{2}^{t,l}=W_{2}^{t-1,l}e^{-\frac{\text{dt}}{\tau_{\text{w}}}}+\eta^{l}S^{t,l-1}\left(\rho \left(U^{t,l}\right)+\beta^{l}\right), \end{array}$$ (8)

where dt denotes the time-step length and τ_w_ is the synaptic time constant. The first term introduces exponential decay to stabilize the weight dynamics, while the second term implements local correlation-based adaptation. In this way, $W_{2}^{l}$ captures pre-synaptic-post-synaptic activity correlation through a local update that depends only on pre- and post-synaptic activities. Crucially, the synaptic weight update itself does not directly use the global loss, which preserves the local nature of Hebbian adaptation. However, as will be described in Section 3.3, the meta-parameters governing this local rule-namely η^*l*^ and β^*l*^ can be globally optimized to align the local learning behavior with the task objective. This two-level design allows the Hebbian channel to maintain its local update dynamics while still benefiting from global task guidance.

#### 3.2.3 SBP-based local-feedback plasticity

SBP provides a local-feedback learning channel. Different from STBP, SBP does not rely on explicit gradient backpropagation through the full network. Instead, SBP constructs local learning signals from layer-wise activity or weight variation, which makes the update more local while still preserving a feedback-driven adjustment effect.

In this work, we use a modified SBP form. Instead of using STDP-derived weight changes as in the original formulation (Zhang et al., 2021), the present implementation uses Hebbian-induced weight variation as the local teaching signal.


$$\begin{array}{l} \Delta W_{2}^{t,l}=W_{2}^{t,l}-W_{2}^{t-1,l} \end{array}$$ (9)

Equation 9 denotes the Hebbian weight change at layer *l* and time step *t*. The SBP channel $W_{3}^{l}$ is updated by


$$\begin{array}{l} W_{3}^{t,l}=W_{3}^{t-1,l}e^{-\frac{\text{dt}}{\tau_{\text{w}}}}+H\left(\Delta W_{2}^{t,l+1},\Delta W_{2}^{t,l}\right), \end{array}$$ (10)

where the local feedback mapping *H*(·, ·) is defined as


$$\begin{array}{l} H\left(A,B\right)=\left(\lambda_{\text{f}}E_{\text{diag}}\left(V+\lambda_{\text{p}}\delta_{n}\left(R\left(A\right)\right)\right)\right)\cdot B, \end{array}$$ (11)

with


$$\begin{array}{l} \delta_{n}\left(x\right)=\frac{x}{\sum_{i}x_{i}}. \end{array}$$ (12)

Here, λ_f_ and λ_p_ are fraction factors in the range [0.1, 1], *E*_diag_(*x*) maps a vector *x* to a diagonal matrix, *V* is an all-ones vector, and *R*(·) denotes the row-wise sum of a matrix.

In this formulation, the higher-layer Hebbian variation $\Delta W_{2}^{t,l+1}$ is first compressed into a layer-level modulation signal through *R*(·) and normalized by δ_*n*_(·). The resulting signal is then converted into a diagonal modulation matrix and applied to the current-layer Hebbian variation $\Delta W_{2}^{t,l}$. In this way, $W_{3}^{l}$ receives a local feedback signal constructed from neighboring-layer plastic changes, without requiring explicit global gradient propagation.

Overall, the three channels play different roles: STBP provides error-driven global supervision, Hebbian learning provides local correlation-based adaptation, and SBP provides local feedback adjustment. These three mechanisms are introduced separately here, and the next subsection describes how the three channels are coordinated within the proposed method.

### 3.3 MPSL method

**Figure 2:**
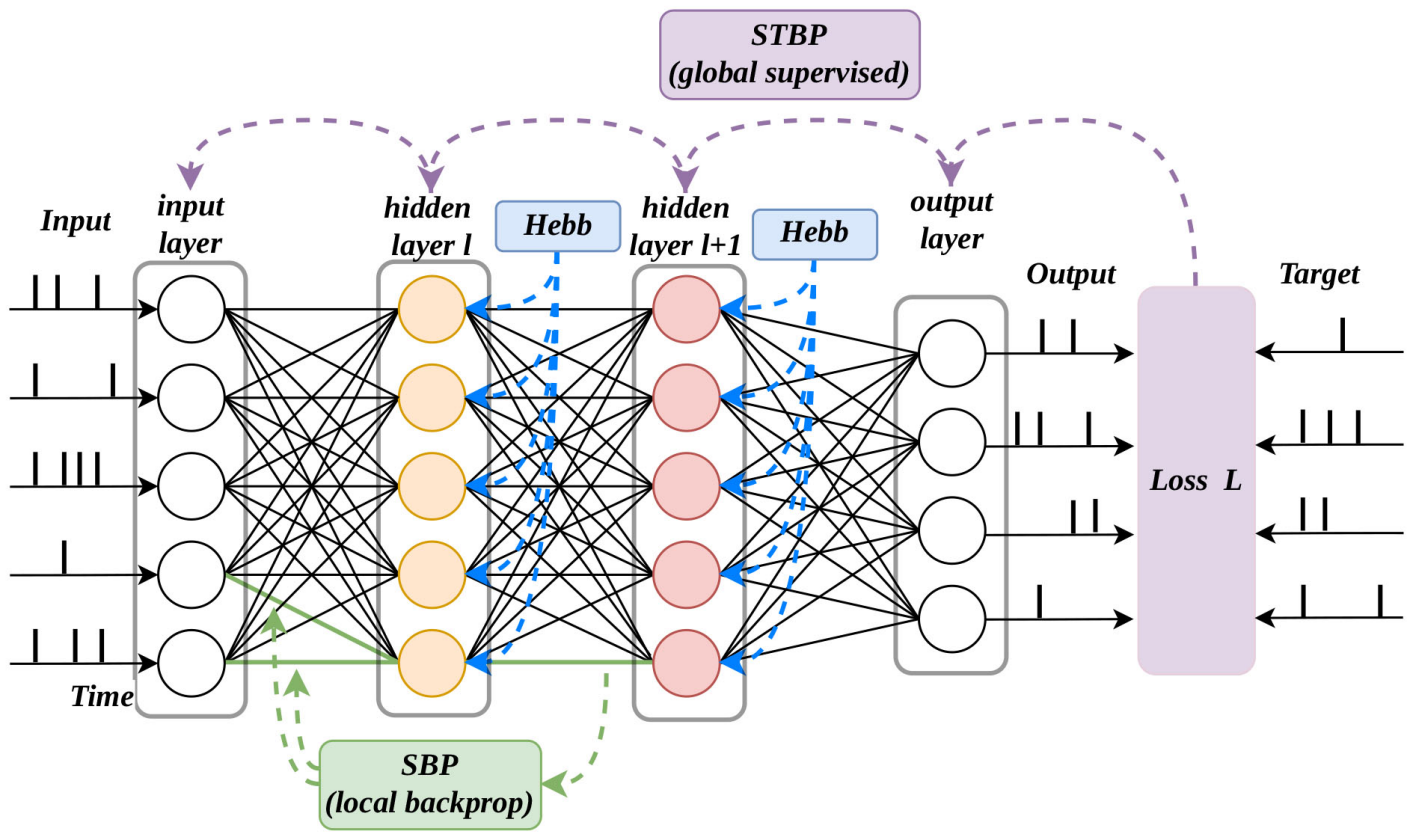
The overall learning process of the proposed MPSL method. It coordinates three distinct learning channels, fusing forward activity correlation, local feedback, and global backpropagation to jointly govern the state evolution of the shared membrane potential.

Based on the neuron model in Section 3.1 and the three plasticity mechanisms introduced in Section 3.2, we next describe how the three learning channels are coordinated in the proposed MPSL method, as illustrated in Figure 2. The main idea of MPSL is to preserve the distinct update pathway of each mechanism during training, while allowing all channels to jointly contribute to a shared membrane potential update.

For the *l*-th layer, MPSL assigns one synaptic component to each learning mechanism, denoted by $W_{1}^{l}$, $W_{2}^{l}$, and $W_{3}^{l}$, corresponding to STBP, Hebbian plasticity, and SBP, respectively. During training, the synaptic input is computed by aggregating the contributions of all channels, as formulated in Equation 13:


$$\begin{array}{l} I^{t,l}=f\left(\overset{n}{\underset{i=1}{\sum }}\lambda_{i}W_{i}^{l},S^{t,l-1}\right), \end{array}$$ (13)

where *n* is the number of learning mechanisms and λ_*i*_ is a learnable modulation coefficient. In this work, *n* = 3. This form keeps the three learning channels separate in the update process, while allowing the three channels to jointly shape a unified membrane potential accumulation and spike response.

**Figure 3:**
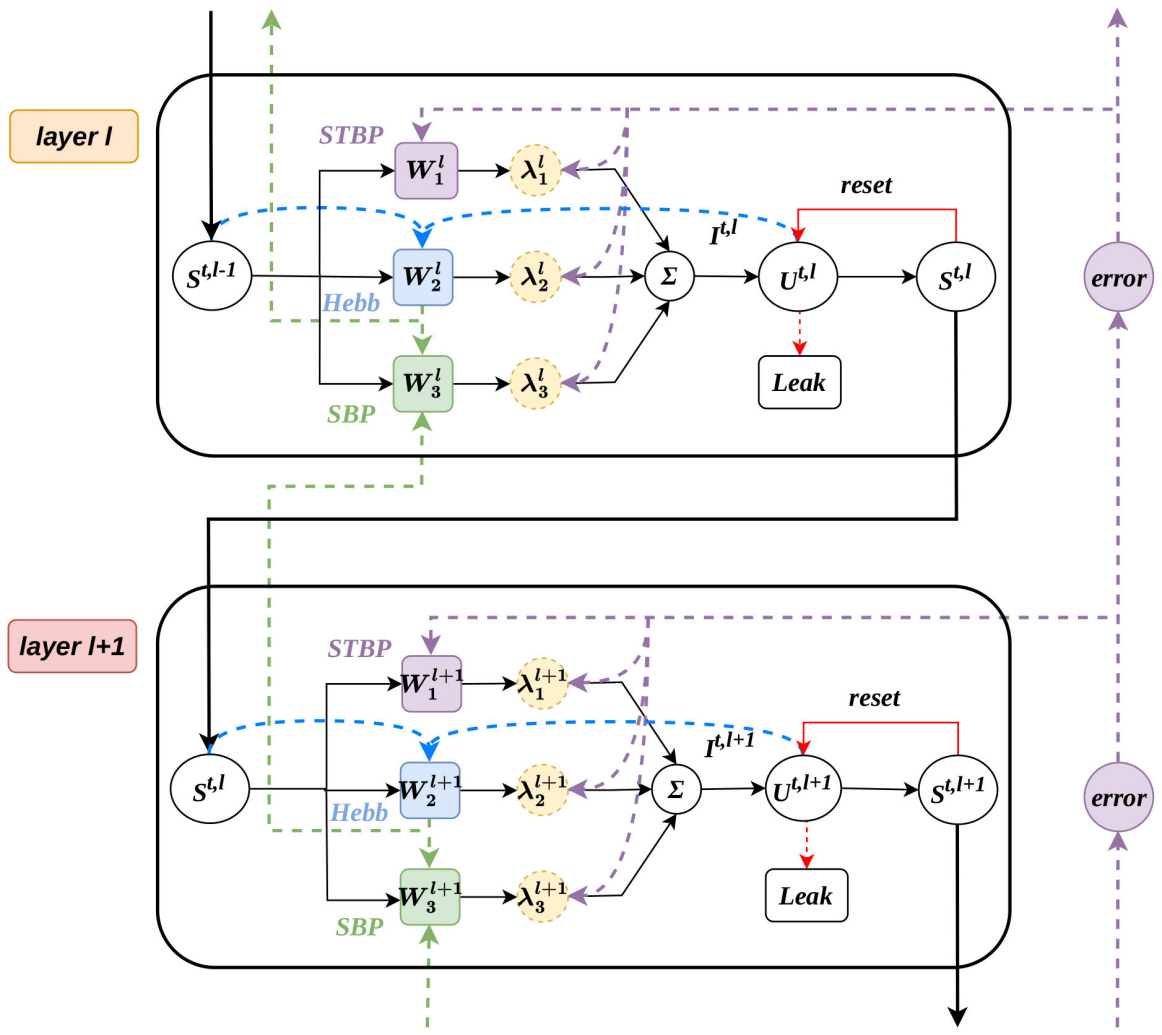
Detailed state changes of the neurons and the multi-synapse parameter update process. Distinct update trajectories for each synapse reveal how local correlations, weight-based feedback, and surrogate gradients jointly drive the membrane potential dynamics.

During training, the three channels are updated through different pathways within a coordinated learning process, as detailed in Figure 3. Hebbian plasticity updates $W_{2}^{l}$ online during the forward process according to local activity correlation. SBP then updates $W_{3}^{l}$ by using local feedback signals constructed from Hebbian weight variation. After all time steps are processed, STBP updates $W_{1}^{l}$ through surrogate gradient based backpropagation. Therefore, the three mechanisms are not simply placed in parallel. Instead, the three channels interact during training and regulate membrane potential dynamics from different perspectives.

In addition to optimizing its own synaptic component, the global error signal from STBP is also used to optimize the learnable parameters involved in the local channels, including η^*l*^ and β^*l*^ in Hebbian plasticity, as well as λ_f_ and λ_p_ in SBP. The modulation coefficients λ_*i*_ are also optimized during training. In this way, global supervision is introduced into the local plasticity channels, which improves coordination among the three mechanisms and makes the overall learning process more stable.

Importantly, optimizing these meta-parameters using the global error does not compromise the local nature of the synaptic weight update in Equation 8. The Hebbian weight $W_{2}^{l}$ is still updated online based solely on local pre- and post-synaptic activities, without direct access to the global loss. The global error only adjusts how the local rule behaves (i.e., its update strength and direction), rather than directly modifying the weight update itself. This separation between local weight dynamics and global meta-parameter optimization constitutes a key design principle of MPSL, enabling the Hebbian channel to preserve its biological plausibility while benefiting from task-level coordination.

To further clarify the update scheduling, Algorithm 1 summarizes the overall training procedure of MPSL. For each time step, the network first performs spike propagation and updates the Hebbian channel in a layer-wise forward manner. Then, the SBP channel is updated in a reverse layer order based on local feedback constructed from Hebbian plastic changes. After all time steps are completed, the output loss is computed and STBP performs gradient-based optimization. This schedule allows local plasticity, local feedback, and global supervision to operate within one unified training process.

Algorithm 1 Training procedure of MPSL. 
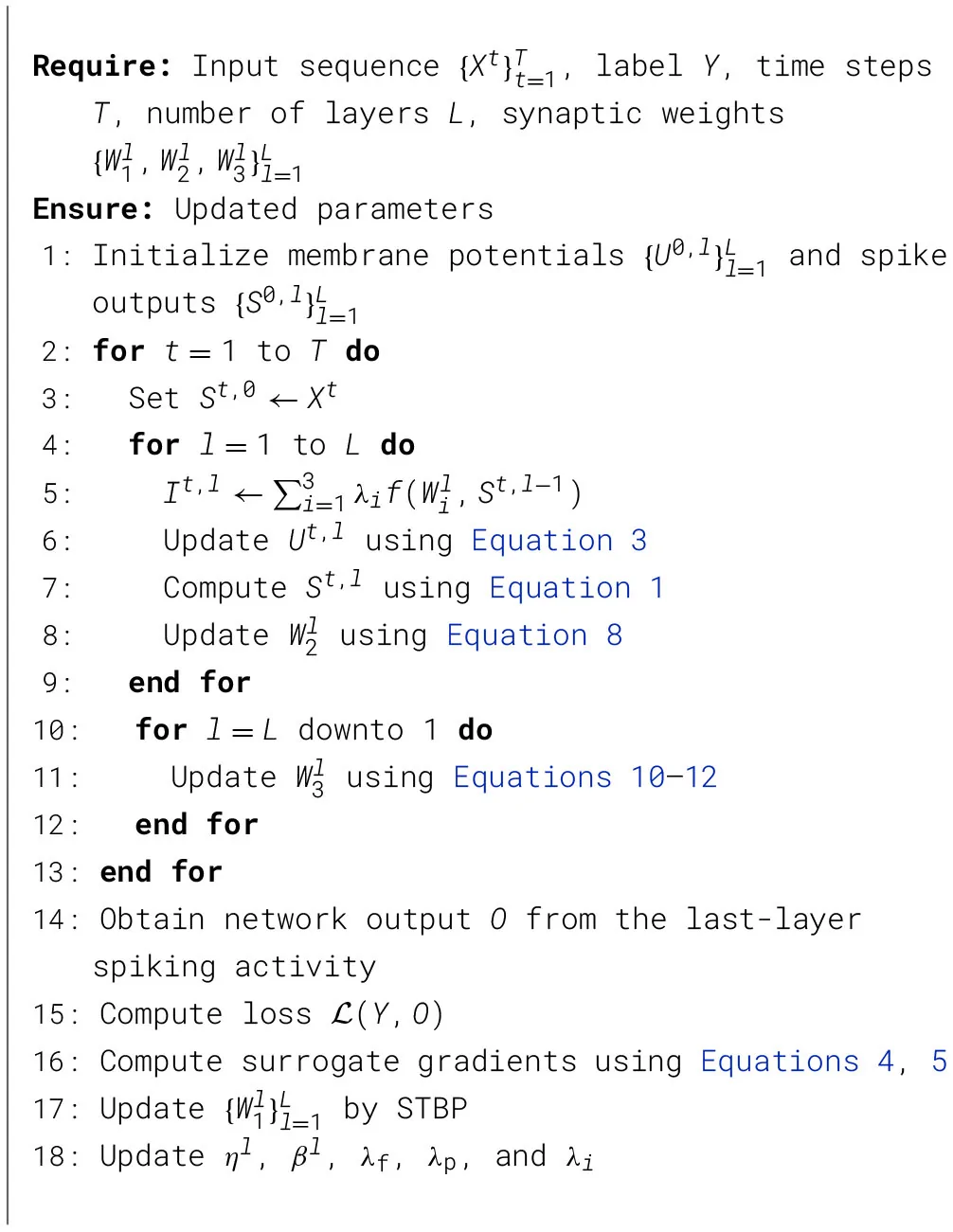

Hebbian plasticity provides local correlation-driven adaptation, SBP reuses local plastic changes to form a feedback-based update pathway, and STBP supplies global error-driven optimization. As a result, the three channels contribute complementary learning signals during training. This coordinated process improves temporal representation and strengthens the adaptability of the network.

Although separate synaptic components are maintained during training, MPSL does not increase inference-time parameter cost. After training, the three synaptic components are linearly merged into a single effective weight, as given in Equation 14:


$$\begin{array}{l} W^{l}=\overset{n}{\underset{i=1}{\sum }}\lambda_{i}W_{i}^{l}. \end{array}$$ (14)

Therefore, the deployed model keeps the same parameter form as a conventional single-weight SNN, while benefiting from multiple coordinated learning signals during training.

The proposed method is not limited to the specific combination of STBP, Hebbian plasticity, and SBP. The present implementation uses these three mechanisms as representative instances, but the MPSL design can be extended to other learning rules as long as the corresponding update signals can be mapped to the shared synaptic input and membrane potential update. This property gives MPSL good flexibility as a general multi-mechanism learning method for SNNs.

## 4 Experiments

We evaluate the proposed MPSL method on multiple tasks and data modalities. We further evaluate robustness under noise and cropping perturbations, and conduct ablation studies on learnable fusion coefficients and feature representations. These experiments verify the effectiveness, generality, and robustness of the proposed method.

### 4.1 Experimental settings

#### 4.1.1 Datasets

For static image classification, we use MNIST, Fashion-MNIST, and CIFAR-10, which range from simple grayscale digits to complex natural scenes, with standard train/test splits of 60k/10k (MNIST, Fashion-MNIST) and 50k/10k (CIFAR-10). For dynamic event-based recognition, we adopt DVS-Gesture, a widely used benchmark collected by neuromorphic vision sensors. DVS-Gesture consists of gesture recordings from 29 subjects performing 11 different hand gestures under three illumination conditions, and we follow the standard subject-independent train/test split. For speech-based recognition, we use the Spiking Heidelberg Digits (SHD) and Google Speech Commands (GSC) datasets. SHD contains approximately 10,420 spoken digit samples (0–9 in English and German, 20 classes) encoded as 700-channel cochlear spike trains. GSC (v0.02) contains approximately 105,000 short utterances across 35 keyword classes, converted to spike trains using the same cochlear encoding. We also used the EEG dataset DEAP, which contains peripheral EEG and physiological signals recorded from 32 participants while watching 40 1-min music video clips. Each test is annotated on four emotional dimensions-valence, arousal, dominance, and liking-using a 1–9 self-assessment scale.

#### 4.1.2 Implementation details

All experiments are implemented using PyTorch (version 2.0.1) with CUDA 11.8 and conducted on a server equipped with NVIDIA RTX 4090 GPUs. We follow consistent training settings across all datasets unless otherwise stated. The details of the simulation time steps, training epochs, batch size, and some neuronal hyperparameters are summarized in Table 1.

**Table 1:** Parameter settings on different learning tasks.

Parameters	Descriptions	MNIST	FMNIST	CIFAR10	DVS-Gesture	SHD	GSC
Batch size	–	100	100	50	32	256	128
*T*	Time steps	8	8	8	8	100	100
τ_*w*_	Time constant	40	40	200	200	10	10
*V*_*th*_	Threshold	0.3	0.4	0.5	0.4	1.0	1.0
*N*	Training epochs	100	200	150	200	200	150

**Network architectures**. We adopt the same network structures as in prior work to ensure fair comparisons. For the MNIST and Fashion-MNIST datasets, we use a five-layer CNN with the structure: [input--128C3--AP2--256C3--AP2--256C3--AP2--512FC--10]. For the DVS-Gesture dataset, we employ a nine-layer CNN: [input--64C3S2--BN--128C3S1--BN--2 56C3S1--BN--256C3S1--BN--256C3S1--BN--256C3S1--AP2--800FC--512FC--11FC]. Batch normalization (BN) is applied to convolutional layers following (Fang et al., 2021). For the CIFAR-10 dataset, we adopt a nine-layer CNN following the CIFARNet structure (Wu et al., 2019). For the SHD and GSC speech datasets, we adopt the same feedforward SNN backbone as in SNNdelays (Hammouamri et al., 2024) to ensure a fair comparison. Specifically, SHD uses a two-layer feedforward SNN with 256 hidden neurons per layer, taking 700-channel cochlear spike trains as input and producing 20-class outputs. GSC uses a three-layer feedforward SNN with the same architectural design principle, taking cochlear-encoded spike trains as input and producing 35-class outputs. The MPSL multi-plasticity framework is applied on top of these backbones during training, while the inference-time model remains a standard single-weight SNN.

**Parameter initialization and constraints**. The fusion coefficients are initialized with a strong prior favoring the global gradient channel: λ_1_ = 1, λ_2_ = 0.1, and λ_3_ = 0.01, corresponding to the STBP, Hebbian, and SBP channels, respectively. All three coefficients are constrained to be strictly positive during training using the clamp function. The Hebbian rule parameters η^*l*^ and β^*l*^ are initialized by sampling from a uniform distribution U(0,0.01). The SBP fraction factors λ_*f*_ and λ_*p*_ are initialized to 0.3 and 0.7, respectively, and both are constrained to the range [0.1, 1] using the clamp function.

### 4.2 Classification performance

We compare the classification performance of our proposed MPSL method with several representative learning algorithms for SNNs. The evaluation is conducted on the EEG dataset DEAP, the image and event-based benchmarks MNIST, CIFAR-10, and DVS-Gesture, as well as the speech datasets SHD and GSC.

We evaluate MPSL on the EEG dataset DEAP under both 2-class and 3-class emotion recognition settings. As shown in Table 2, our method achieves the best performance in both settings. Specifically, in the 2-class setting, MPSL reaches 92.36% on valence and 92.76% on arousal. In the 3-class setting, MPSL achieves 89.95% on valence and 90.79% on arousal. Compared with existing methods, our method maintains clear advantages on both evaluation protocols, further verifying its effectiveness on EEG signals.

**Table 2:** Comparison with existing methods on the DEAP dataset under 2-class and 3-class settings.

Dataset	Method	Plasticity	Class	Valence (%)	Arousal (%)
DEAP	Transfer learning (Yan et al., 2022)	Global	2	82.75	84.22
	SNN+IR (Xu et al., 2024)	Global	2	61.15	53.86
	EEGNet (Gong et al., 2023)	Global	2	81.14	76.82
	SGLNet (Gong et al., 2023)	Global	2	90.01	89.41
	SCNN (Islam et al., 2021)	Global	3	70.23	70.25
	DH-SNN (Zheng et al., 2024)	Global	3	77.46	80.23
	BSNN (Sun et al., 2025)	Global	3	81.03	82.60
	**MPSL (Ours)**	Hybrid	2	**92.36**	**92.76**
		Hybrid	3	**89.95**	**90.79**

Bold values indicate the best-performing result among all compared methods for each setting.

On image and event-based benchmarks, as shown in Table 3, our method consistently achieves top performance, reaching 99.52% on MNIST, 92.12% on CIFAR-10, and 97.22% on DVS-Gesture. Compared with representative SNN learning methods such as STDP, SBP, STBP, and HGLL, MPSL shows clear improvements on both static visual inputs and dynamic event streams. These results further demonstrate the effectiveness of integrating complementary learning mechanisms for visual and event-driven recognition tasks.

**Table 3:** Comparison with existing methods on the MNIST, CIFAR-10, and DVS-Gesture datasets.

Dataset	Method	Plasticity	Timestep	Accuracy (%)
MNIST	STDP (Diehl and Cook, 2015)	Local	350	95.00
	SBP (Zhang et al., 2021)	Hybrid	10	95.14
	STBP (Wu et al., 2018)	Global	30	98.89
	HGLL (Wu et al., 2022)	Hybrid	8	99.50
	**MPSL (Ours)**	Hybrid	8	**99.52**
CIFAR-10	STBP (Wu et al., 2018)	Global	8	90.55
	HGLL (Wu et al., 2022)	Hybrid	8	91.08
	**MPSL (Ours)**	Hybrid	8	**92.12**
DVS-Gesture	SBP (Zhang et al., 2021)	Hybrid	10	84.76
	STBP (Wu et al., 2018)	Global	8	96.21
	HGLL (Wu et al., 2022)	Hybrid	8	97.01
	**MPSL (Ours)**	Hybrid	8	**97.22**

For STDP on MNIST, the timestep 350 is calculated based on 1 ms per step over a 350 ms input duration, consistent with the Poisson encoding in Diehl and Cook (2015). The CIFAR-10 baseline result of STBP is reproduced under the same conditions based on the authors' released code. Bold values indicate the best-performing result among all compared methods for each setting.

Finally, on speech benchmarks, our method continues to show strong performance. As shown in Table 4, MPSL achieves 95.10% on SHD and 95.14% on GSC, which are both higher than the compared prior SNN methods. These results further demonstrate the overall superior generalization ability of our method across different input modalities.

**Table 4:** Comparison with prior SNN methods on the SHD and GSC speech datasets.

Dataset	Method	Plasticity	Timestep	Accuracy (%)
SHD	RadLIF (Bittar and Garner, 2022)	Global	100	94.62
	TC-LIF (Zhang et al., 2024)	Global	-	88.91
	TA-SNN (Yao et al., 2021)	Global	100	91.08
	SNNdelays (Hammouamri et al., 2024)	Global	100	93.13
	SpikeSCR (Wang et al., 2025)	Global	100	94.13
	**MPSL (Ours)**	Hybrid	100	**95.10**
GSC	RadLIF (Bittar and Garner, 2022)	Global	100	94.51
	TC-LIF (Zhang et al., 2024)	Global	-	94.84
	Speech2spike (Stewart et al., 2023)	Global	200	88.50
	SNNdelays (Hammouamri et al., 2024)	Global	100	93.32
	SpikeSCR (Wang et al., 2025)	Global	100	94.29
	**MPSL (Ours)**	Hybrid	100	**95.14**

Results of SNNdelays and SpikeSCR are reproduced under the same conditions based on the authors' released code. Bold values indicate the best-performing result among all compared methods for each setting.

As summarized in Table 5, MPSL introduces negligible extra trainable overhead during training and matches the backbone STBP parameter count at inference.

**Table 5:** Parameter statistics of STBP and MPSL across different datasets.

Dataset	STBP	MPSL trainable	Online *W*_2_/*W*_3_ states	MPSL deployment
MNIST	2.134 M	2.141 M	0.134 M	2.134 M
DVS-Gesture	3.379 M	3.382 M	1.650 M	3.379 M
SHD	0.214 M	0.214 M	0.213 M	0.214 M
GSC	1.231 M	1.231 M	1.228 M	1.231 M

STBP parameters denote the trainable parameters of the conventional SNN backbone. MPSL trainable parameters include the globally optimized synaptic weights and a small number of learnable rule-control parameters, whereas the online *W*_2_ and *W*_3_ states are maintained and updated by the local Hebbian and SBP rules rather than by the global optimizer. After training, the three synaptic components are merged into a single effective weight, and the additional plasticity states are discarded for inference. Thus, the deployment parameter count remains comparable to that of the corresponding backbone.

Overall, the consistent improvements across EEG, image, event-based, and speech datasets validate the effectiveness of integrating complementary learning mechanisms and highlight the robustness and generalization ability of our method under diverse data conditions.

### 4.3 Robustness evaluation

#### 4.3.1 Noise robustness

To evaluate the robustness of our method under input perturbations, we conduct inference under two commonly studied noise types: Gaussian noise and salt-and-pepper noise. Gaussian noise adds continuous-valued fluctuations to the input intensities, while salt-and-pepper noise randomly corrupts a proportion of pixels to either minimum or maximum intensity, simulating spike-like disturbances. Both types of noise are applied under the same feedforward architecture to isolate the influence of the learning method.

**Figure 4:**
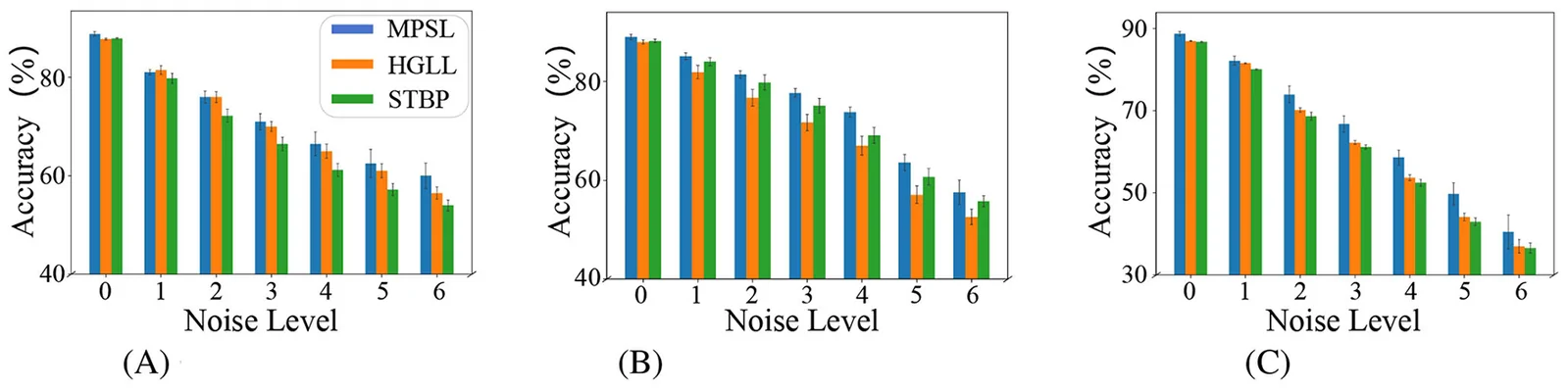
Performance under varying levels of Gaussian noise, salt-and-pepper noise, and input cropping, where the levels correspond to increasing variance for Gaussian noise, increasing noise amount for salt-and-pepper noise, and decreasing crop size for input cropping. Results are averaged over 5 runs, and the error bars represent performance variability across multiple runs. **(A)** Gaussian noise; **(B)** Salt-and-pepper noise; **(C)** Cropping.

As shown in Figures 4A, B, our proposed MPSL consistently demonstrates superior accuracy under increasing noise levels compared to both the single-mechanism baseline (STBP) and the existing hybrid baseline (HGLL). The outperformance over STBP confirms that integrating complementary learning rules fundamentally enhances resilience to input corruption. Furthermore, the advantage over HGLL suggests that our specific multi-branch synaptic design extracts more robust feature representations than conventional hybrid methods.

#### 4.3.2 Cropping robustness

To further evaluate the resilience of the model under partial occlusion or information loss, we apply center cropping to input images with different levels of severity (Hendrycks and Dietterich, 2019; Djolonga et al., 2021). Specifically, the original images are cropped to smaller central patches, simulating increasing degrees of spatial information reduction. As shown in Figure 4C, our method consistently outperforms baseline models across all cropping levels and maintains significantly higher accuracy even under severe information loss. These results highlight the enhanced spatial redundancy and robustness of the proposed multi-mechanism method, which can better retain discriminative features despite partial input loss.

### 4.4 Ablation study

#### 4.4.1 Effect of learnable fusion coefficients

To further investigate the importance of dynamically tuning the fusion coefficients that govern the interaction strength among learning mechanisms, we perform an ablation study on the Fashion-MNIST and DVS-Gesture datasets, using the same five-layer CNN and nine-layer CNN architectures as in the main experiments, respectively. We evaluate multiple settings: (1) **Fixed coefficients**: all three fusion coefficients are set to equal static values (λ_1_ = λ_2_ = λ_3_ = 1/3) throughout training; (2) **Learnable coefficients**: the fusion coefficients are jointly optimized with the network parameters; (3) **Frozen learned initialization**: the fusion coefficients are first trained to convergence in a separate fully adaptive run; the learned coefficient values are then fixed, and the network weights are reinitialized and retrained from scratch with these frozen coefficients.

**Figure 5:**
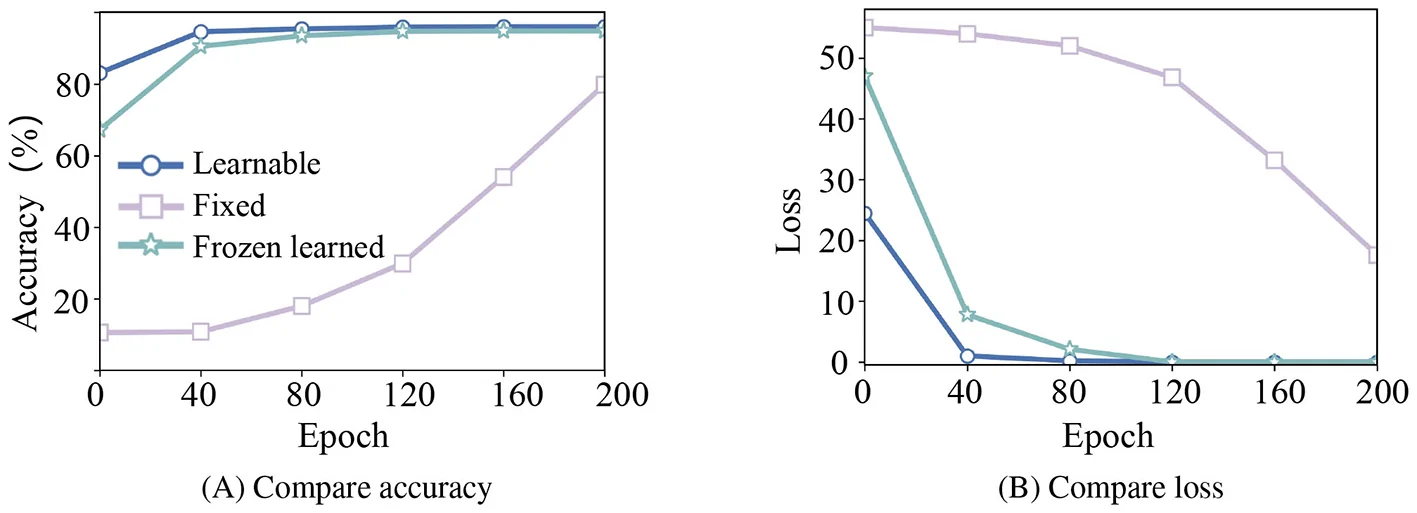
Comparison of training accuracy and loss under different fusion coefficient strategies: fixed, learnable, and frozen learned. **(A)** Compare accuracy; **(B)** Compare loss.

As shown in Figure 5, the learnable setting yields the highest performance on Fashion-MNIST, indicating that continuous adaptation of mechanism contribution is vital for aligning with task demands and input statistics. Notably, the frozen learned setting substantially outperforms the fully fixed one on the test set, implying that the learned coefficients capture a favorable inductive bias even when the network is retrained from scratch.

To further dissect the contribution of each fusion coefficient, we extend the ablation to partially adaptive configurations in which only a subset of the coefficients is learnable while the rest are fixed. Specifically, we evaluate settings with only one learnable coefficient (λ_1_, λ_2_, or λ_3_), settings with two learnable coefficients (λ_1_+λ_2_, λ_1_+λ_3_, or λ_2_+λ_3_), and the fully adaptive setting with all three coefficients learnable. All experiments are conducted on Fashion-MNIST and DVS-Gesture using the same backbone architecture and training protocol. The results are summarized in Table 6.

**Table 6:** Ablation of adaptive fusion coefficients on Fashion-MNIST and DVS-Gesture.

Setting	FMNIST (%)	DVS-gesture (%)
Fixed	80.39	90.11
Frozen learned	92.77	96.98
Only λ_1_	91.94	96.53
Only λ_2_	91.87	95.84
Only λ_3_	91.22	95.27
λ_1_+λ_2_	92.90	96.88
λ_1_+λ_3_	93.17	96.83
λ_2_+λ_3_	92.56	95.18
**λ_1_+λ_2_+λ_3_ (Full)**	**93.72**	**97.22**

All results are test-set accuracy. Each row indicates which fusion coefficients are learnable; the remaining coefficients are fixed at their initial values. The Fixed setting uses λ_1_ = λ_2_ = λ_3_ = 1/3. The Frozen Learned setting fixes coefficients obtained from a prior fully adaptive run and retrains the network from scratch. Bold values indicate the best-performing result among all compared methods for each setting.

As shown in Table 6, the fully adaptive setting (λ_1_+λ_2_+λ_3_) achieves the best performance on both datasets (93.72% on FMNIST and 97.22% on DVS-Gesture), confirming that adaptive coordination of all three channels is optimal. Among the single-coefficient settings, allowing only λ_1_ to be learnable yields the highest accuracy (91.94/96.53%), consistent with the dominant role of the global STBP channel. However, every single-coefficient setting underperforms the fully adaptive model. Among the two-coefficient settings, λ_1_+λ_3_ achieves the strongest result (93.17/96.83%), closely followed by λ_1_+λ_2_ (92.90/96.88%), while λ_2_+λ_3_ without the global channel performs notably worse (92.56/95.18%). These results demonstrate that while the global STBP channel is the primary driver, the local Hebbian and SBP channels provide complementary and non-redundant contributions that are essential for reaching the full performance of MPSL.

**Figure 6:**
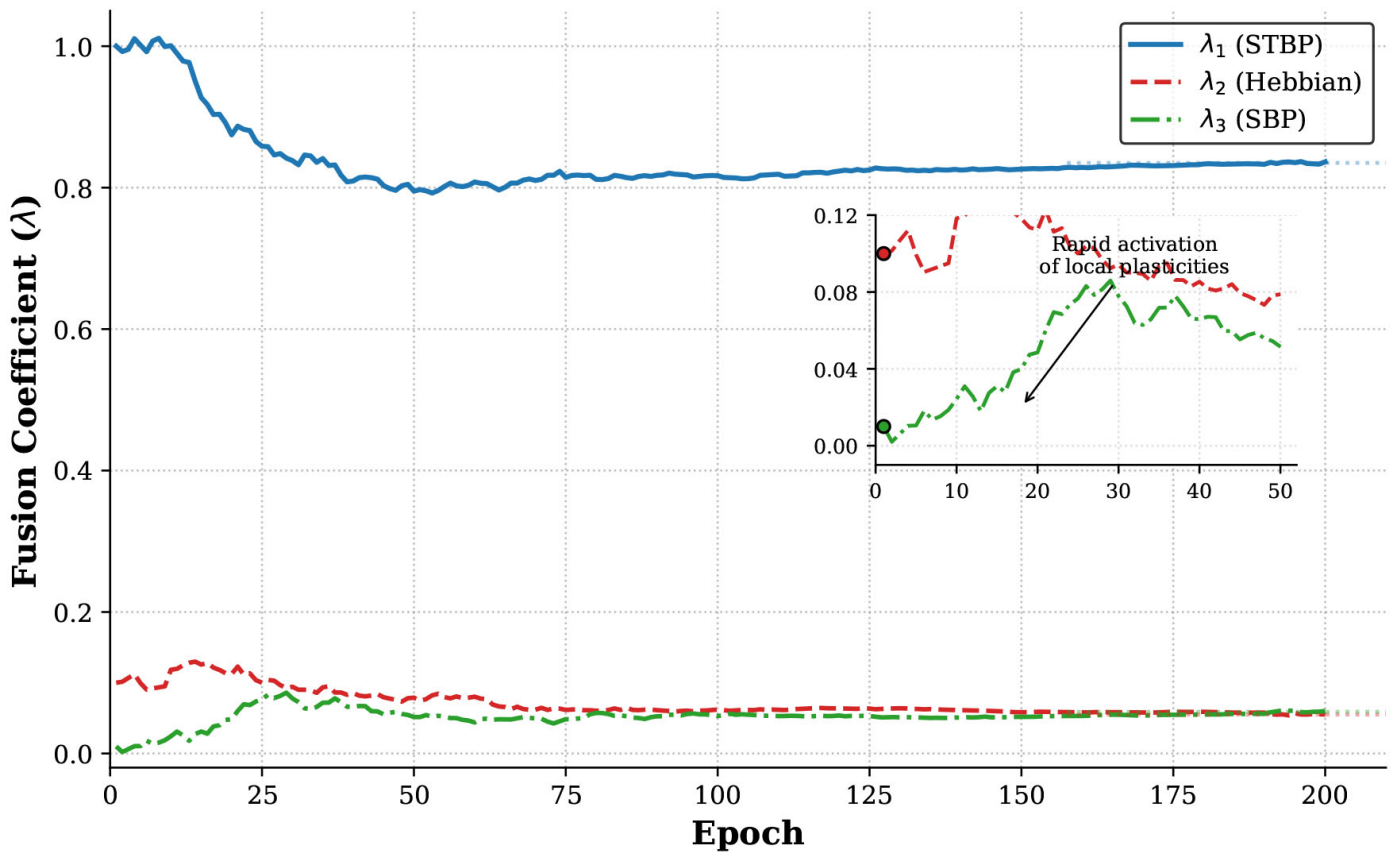
Evolution of learnable fusion coefficients λ_1_ (STBP), λ_2_ (Hebbian), and λ_3_ (SBP) during training on DVS-Gesture. The inset zooms into the first 50 epochs to highlight the early rapid activation of local plasticity channels. After convergence, all three coefficients stay non-zero, verifying sustained synergy across the three learning pathways rather than degeneration to the pure STBP baseline.

To verify dynamic synergy across learning pathways, Figure 6 plots the evolution of fusion coefficients on DVS-Gesture. λ_1_ gradually settles as the dominant global gradient channel, while λ_2_ and λ_3_ activate rapidly in early training and maintain stable non-zero values at convergence, demonstrating persistent contributions from local Hebbian and SBP plasticity.

#### 4.4.2 Effect of mechanism ablation on feature representations

To gain a deeper understanding of how different learning mechanisms affect the internal representations of the spiking neural network, we conduct a feature visualization experiment using t-SNE. We select 500 test samples from the Fashion-MNIST dataset and extract the membrane potentials from the penultimate layer, resulting in 128-dimensional feature vectors. These features are then projected into a two-dimensional space for visualization.

**Figure 7:**
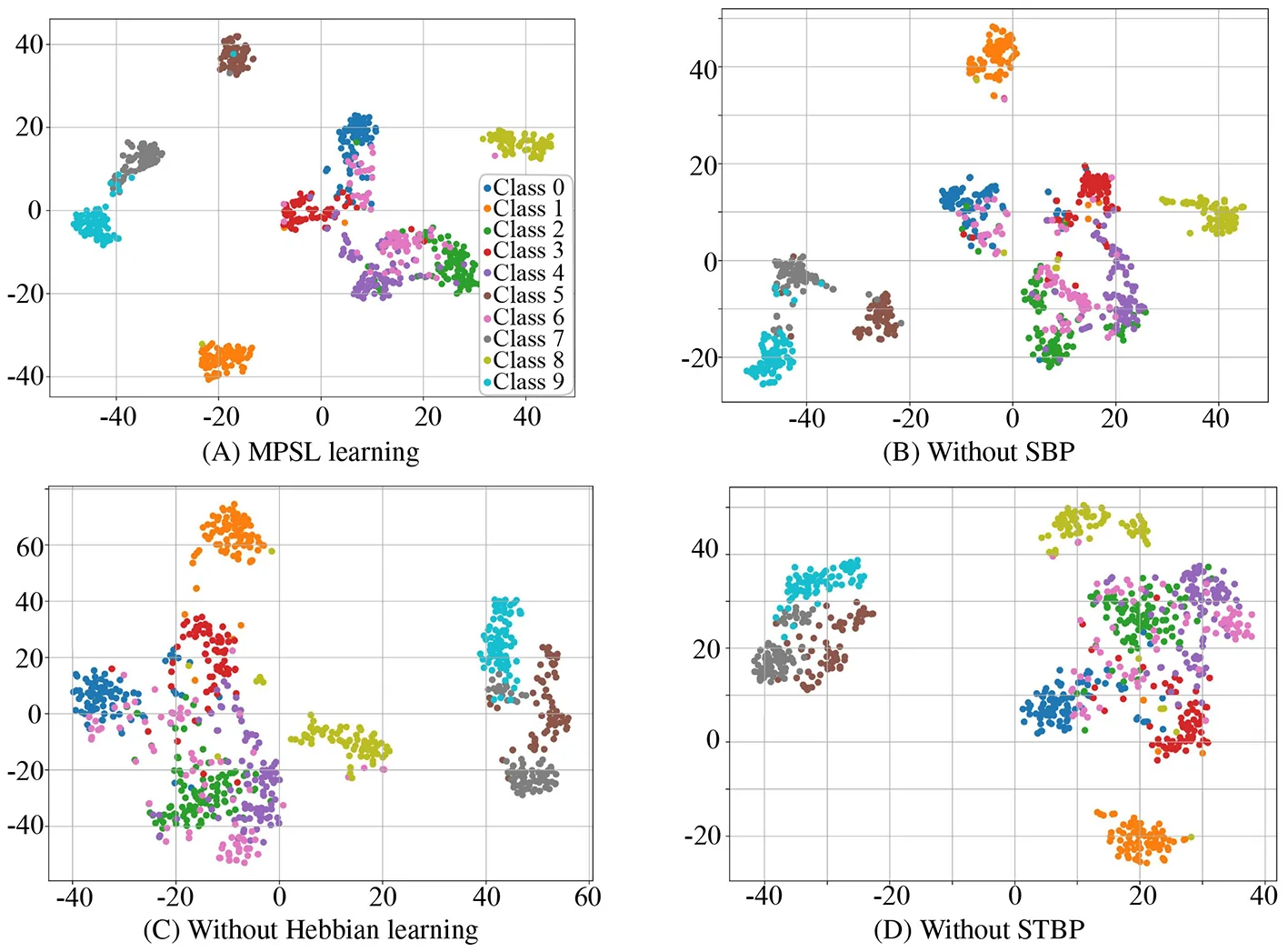
t-SNE visualizations of internal feature representations from the penultimate layer under four training settings: the full model and three ablated variants, each missing one learning mechanism. **(A)** MPSL learning; **(B)** Without SBP; **(C)** Without Hebbian learning; **(D)** Without STBP.

We compare four training configurations: the full model incorporating STBP, Hebbian learning, and SBP, and three ablated versions, each omitting one of the mechanisms. The visualization results, shown in Figure 7, reveal that the full model produces more compact and clearly separated clusters, indicating strong class-wise discrimination and intra-class consistency. In contrast, the ablated variants exhibit more scattered or overlapping feature distributions. These visualizations suggest that the integration of multiple learning rules enriches the network's representational capacity, supporting better generalization and robustness.

#### 4.4.3 Necessity of multi-plasticity synergy and generality of the framework

To verify that the performance gain of MPSL arises from genuine synergy among multiple plasticity mechanisms rather than from any single component, and to substantiate the claimed generality of the framework, we conduct two additional sets of controlled experiments.

**Mechanism combination analysis**. We first compare single-mechanism, pairwise, and full three-mechanism configurations under the same backbone architecture, time steps, optimization protocol, and data splits. The evaluated configurations include STBP only, original SBP only, STBP+Hebbian, STBP+SBP, the existing hybrid method HGLL, and the complete STBP+Hebbian+SBP model (MPSL). All methods are evaluated on the static MNIST benchmark and the dynamic DVS-Gesture benchmark to cover both static and dynamic input modalities.

As shown in Table 7, the full three-mechanism MPSL consistently outperforms all single-mechanism and pairwise configurations on both datasets. Specifically, on DVS-Gesture, MPSL achieves 97.22%, compared with 96.21% for STBP only, 84.76% for original SBP, 96.52% for STBP+Hebbian, and 96.18% for STBP+SBP. The pairwise combination STBP+SBP yields improved accuracy relative to vanilla STBP on MNIST, while it delivers slightly degraded performance on DVS-Gesture. In contrast, the STBP+Hebbian combination only delivers performance improvement on DVS-Gesture and underperforms vanilla STBP on MNIST. However, neither pairwise combination reaches the performance of the full three-mechanism model, demonstrating that all three mechanisms contribute non-redundant information. The comparison with HGLL further shows that the proposed three-channel design achieves stronger synergy than existing hybrid formulations.

**Table 7:** Controlled comparison of different mechanism combinations on MNIST and DVS-Gesture.

Method	MNIST (%)	DVS-gesture (%)
STBP only	98.89	96.21
Original SBP only	95.14	84.76
STBP + Hebbian	98.61	96.52
STBP + SBP	98.99	96.18
HGLL	99.50	97.01
**MPSL (STBP** **+** **Hebbian** **+** **SBP)**	**99.52**	**97.22**

Results of Original SBP are reported by Zhang et al. (2021); all other results are obtained by our own implementation under the same experimental protocol. Bold values indicate the best-performing result among all compared methods for each setting.

**Alternative MPSL instantiation**. To demonstrate that MPSL is not tied to the specific combination of STBP, Hebbian plasticity, and modified SBP, we construct an alternative instantiation of the framework in which the Hebbian rule is replaced with an STDP rule and the modified SBP channel is replaced with the original SBP formulation (Zhang et al., 2021). This alternative configuration preserves the same MPSL fusion architecture and adaptive coefficient mechanism, differing only in the specific local plasticity rules assigned to the local channels.

As reported in Table 8, the alternative MPSL instantiation (STBP+STDP+original SBP) achieves 99.03% on MNIST and 97.36% on DVS-Gesture, which is competitive with the original MPSL configuration and substantially higher than the single-mechanism baselines. This result confirms that the MPSL framework is general and extensible: its effectiveness does not depend on a particular choice of local plasticity rules, and other local learning mechanisms can be readily plugged into the same multi-channel fusion architecture.

**Table 8:** Comparison between the original MPSL instantiation and an alternative instantiation using different local plasticity rules.

Method	Local channel 1	Local channel 2	MNIST (%)	DVS-gesture (%)
MPSL (original)	Hebbian	Modified SBP	99.52	97.22
MPSL (alternative)	STDP	Original SBP	99.03	97.36

Both configurations share the same MPSL fusion architecture and adaptive coefficient mechanism.

## 5 Conclusion

In this paper, we propose MPSL, a multi-plasticity synergy learning method for spiking neural networks. The proposed method allows multiple learning mechanisms to operate as separate update channels during training while jointly contributing to a shared membrane potential update. In this work, we instantiate the method with STBP, Hebbian plasticity, and SBP, so that global error-driven optimization, local correlation-based adaptation, and local-feedback learning can be integrated into one unified learning process.

Extensive experiments on EEG, image, event-based, and speech datasets demonstrate the effectiveness and generality of the proposed method. MPSL achieves strong performance across different tasks and data modalities, and also shows better robustness under noise and cropping perturbations. In addition, the ablation results verify the importance of learnable fusion coefficients and show that coordinated interaction among multiple learning mechanisms improves feature representation and overall learning quality.

Overall, this work provides a general and extensible computational foundation for developing more powerful SNNs through the coordinated use of functionally complementary learning signals. We hope the proposed method can serve as a useful basis for future studies on multi-mechanism learning in spiking neural networks.

## Data Availability

Publicly available datasets were analyzed in this study. This data can be found here: http://yann.lecun.com/exdb/mnist/ for the MNIST dataset, https://cave.cs.toronto.edu/kriz/cifar.html for the CIFAR-10 dateset, https://ibm.biz/EventCameraData for the DVS-Gesture dataset, https://zenkelab.org/resources/spiking-heidelberg-datasets-shd/ for the SHD dataset, https://tensorflow.google.cn/datasets/catalog/speech_commands/ for the GSC dataset. The DEAP dataset is available on request via https://www.eecs.qmul.ac.uk/mmv/datasets/deap/.
